# Myeloid sarcoma with *RBM15::MRTFA (MKL1)* mimicking vascular neoplasm

**DOI:** 10.1007/s00428-024-03766-z

**Published:** 2024-02-20

**Authors:** Fatma Gündoğdu, Abbas Agaimy, Selin Aytaç, Volkan Hazar, Ayşegül Üner, Kemal Kösemehmetoğlu

**Affiliations:** 1 Department of Pathology, Hacettepe University Faculty of Medicine, Ankara, Türkiye; 2 Department of Pathology, Erlangen University, Erlangen, Germany; 3 Department of Pediatric Hematology, Hacettepe University Faculty of Medicine, Ankara, Türkiye; 4 Department of Pediatric Oncology, Akdeniz University, Antalya, Türkiye

**Keywords:** Acute megakaryoblastic leukemia, *RBM15::MRTFA(MKL1)* fusion, Myeloid sarcoma, Malignant mesenchymal tumor, Children, Vascular neoplasm

## Abstract

Extramedullary involvement of acute myeloid leukemia (AML), aka myeloid sarcoma, is a rare phenomenon in acute megakaryoblastic leukemia with *RBM15:: MRTFA(MKL1)* fusion, which might mimic non-hematologic malignancies. A 7-month-old infant presented with leukocytosis, hepatosplenomegaly, multiple lymphadenopathies, and a solid mass in the right thigh. Initially, the patient was diagnosed with a malignant vascular tumor regarding the expression of vascular markers from the biopsy of the right thigh lesion that was performed after the inconclusive bone marrow biopsy. The second bone marrow biopsy, which was performed due to the partial response to sarcoma treatment, showed hypercellular bone marrow with CD34 and CD61-positive spindle cell infiltration and > 20% basophilic blasts with cytoplasmic blebs. RNA sequencing of soft tissue biopsy revealed the presence of *RBM15::MRTFA(MKL1)* fusion. Based on these findings, myeloid sarcoma/AML with *RBM15::MRTFA(MKL1)* fusion diagnosis was made. AML with *RBM15::MRTFA(MKL1)* fusion can initially present as extramedullary lesions and might cause misdiagnosis of non-hematologic malignancies.

## Introduction

Acute megakaryoblastic leukemia (AMKL) is an infrequent subtype (3–5%) of acute myeloid leukemia (AML) that is characterized by > 20% blasts with megakaryocytic differentiation recognized with the combination of morphological, immunohistochemical, and cytogenetic features [1]. Acute myeloid leukemia with *RBM15::MRTFA(MKL1)* fusion is a specific type of AMKL that accounts for 10–12% of the cases [1–3]. It is usually seen in infants without trisomy 21 (Down Syndrome) [2]. Extramedullary involvement of AML known as myeloid sarcoma or extramedullary acute myeloid leukemia can occur simultaneously with bone marrow involvement [4–7]. However, it is a rarely described phenomenon in AMKL, which creates diagnostic difficulties [4, 5]. There are a few reported cases of myeloid sarcoma related to AML with *RBM15::MRTFA(MKL1)* fusion in the literature, which mimic non-hematologic malignancies like neuroblastoma [8, 9], hepatoblastoma [10], or small round blue cell tumor [11], thus the differential diagnosis may be challenging.

Here, we present a case of AML with *RBM15::MRTFA(MKL1)* fusion in a 7-month-old infant. The patient had bone marrow involvement as well as a soft tissue mass in the right thigh. The thigh mass was initially misdiagnosed as a malignant vascular tumor. To the best of our knowledge, this is the first reported case of AML with *RBM15::MRTFA(MKL1)* fusion and soft tissue involvement.

## Case presentation

The patient was a 7-month-old male infant whose prenatal and natal history were unremarkable. In a routine clinical visit, hepatosplenomegaly and anemia were detected. The patient was evaluated for hematolymphoid malignancies and neuroblastoma in another hospital but no definitive diagnosis was made and the patient was referred to our hospital. In the complete blood count, anemia (hemoglobin 8.0 g/dL), thrombocytopenia (56,000 × 10^6^/L), leukocytosis (18,500 × 10^6^/L), and lymphocytosis (13,500 × 10^6^/L) were detected. Radiological examinations revealed abdominal, mediastinal, and hilar multiple lymphadenopathies, hepatosplenomegaly, a lung nodule, subcutaneous nodules in the abdominal and thoracal wall, a solid mass in the right thigh anterior to femur between muscle plains, and diffuse increased signal density in the bone marrow. A bone marrow sampling was performed for further evaluation.

**Fig. 1:**
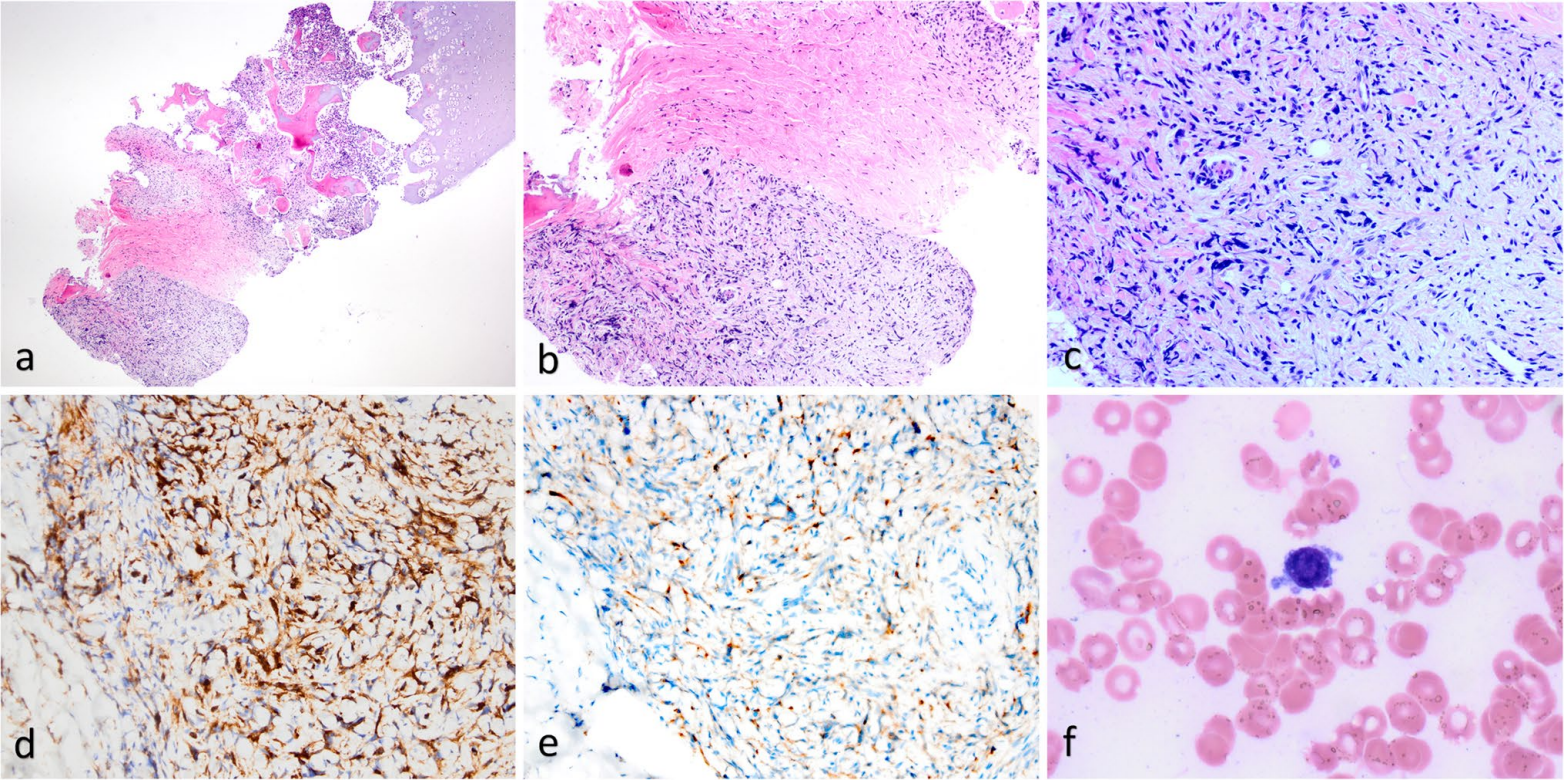
Microscopic examination of the first bone marrow biopsy a) subcortical hypercellular bone marrow (H&E 40x) b) Spindle neoplastic cell infiltration showing crush artifact (H&E 100x) c) Neoplastic cells exhibiting ovoid to spindle-shaped nuclei and irregular nuclear contours (H&E 400x). Neoplastic cells showing immunopositivity for CD34 (d, 200x) and CD61 (e, 200x). f) Scattered blastic cells with fine chromatin, basophilic cytoplasm, and occasional cytoplasmic blebs seen in the aspiration smear (Giemsa 1000x)

In the histological examination of bone marrow biopsy, hypercellular bone marrow with extensive crush artifact and increased reticulin fibrosis mostly representing the subcortical zone was seen (Fig. 1a-b). The limited number of discernable cells mostly had ovoid to spindle-shaped nuclei, irregular nuclear contours, and fine chromatin (Fig. 1c). They showed varied immunopositivity for CD34 (Fig. 1d) and CD61 (Fig. 1e) but the significance of these stainings could not be interpreted optimally due to prominent crush artifacts. Bone marrow aspirate was also suboptimal due to the absence of bone marrow particles. Still, a few blastic cells with fine chromatin, multiple small nucleoli, and occasional cytoplasmic blebs (Fig. 1f) and dysplastic myeloid and erythroid precursor cells were noted. However, it wasn’t possible to reach a definitive diagnosis with this biopsy alone, even though the possibility of AMKL was considered. A biopsy from either the lung or thigh lesions was recommended.

**Fig. 2:**
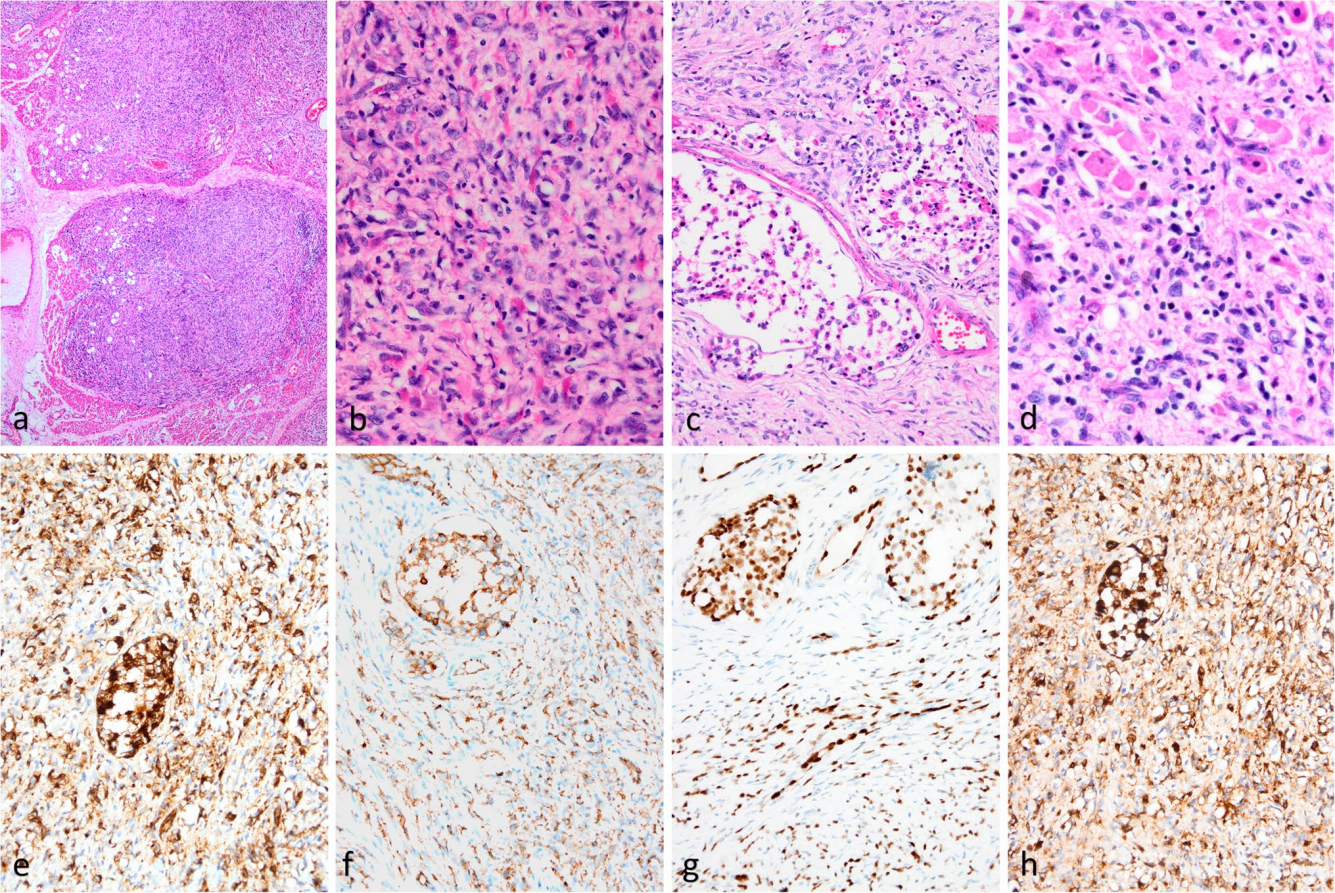
Microscopic examination of the soft tissue lesion showing a multinodular neoplasm infiltrating skeletal muscles (a, 40x) b) High power field view revealed epithelioid cells with blastic chromatin and occasional cytoplasmic vacuoles intermixed with spindle cells and mixed inflammatory cells (400x). c) Epithelioid cells with abundant eosinophilic cytoplasm in the lymphovascular spaces (200x) d) Epithelioid and spindle cells with vesicular chromatin, prominent nucleoli, and irregular nuclear contours as well as accompanying histiocytes and lymphocytes (400x). Neoplastic cells positive for CD34 (d, 200x), CD31 (e, 200x), ERG (f, 200x), and Factor VIIIa (g, 200x), immunohistochemically

Afterward, an initial core biopsy followed by an excisional biopsy from the right thigh lesion was performed. In the microscopic examination of the lesion, a multinodular neoplasm infiltrating adjacent skeletal muscles was seen (Fig. 2a). The neoplasm is comprised of both epithelioid and spindle-looking cells in a fibrotic background. Epithelioid cells with abundant eosinophilic cytoplasm were predominantly observed in the vascular spaces (Fig. 2b). Neoplastic cells exhibited vesicular chromatin, multiple prominent nucleoli, irregular nuclear contours, and increased mitotic activity (Fig. 2c). They were positive for CD34 (Fig. 2d), CD31 (Fig. 2e), ERG (Fig. 2f), and Factor VIII (Fig. 2g), and negative for CD163 immunohistochemically. Based on morphologic and immunophenotypic results a malignant vascular neoplasm most consistent with composite hemangioendothelioma was considered in the foreground of the differential diagnosis. A sample from the soft tissue mass was sent out for the detection of DNA and RNA alterations using next-generation sequencing.

**Fig. 3:**
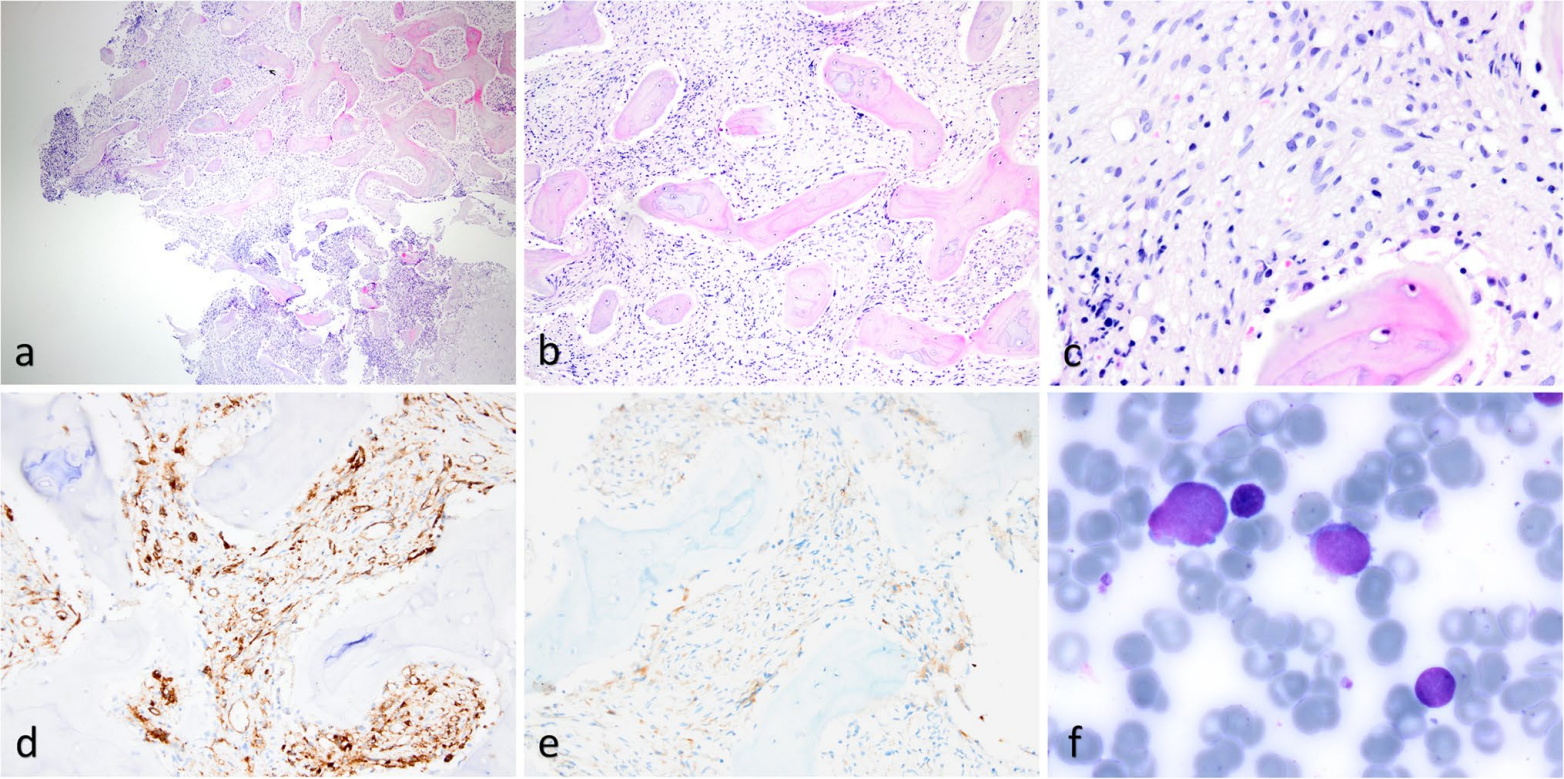
Microscopic examination of the second bone marrow biopsy showing a) fibrotic hypercellular bone marrow (40x) b) with spindle neoplastic cell infiltration (100x) c) neoplastic cells exhibiting ovoid to spindle-shaped nuclei, irregular nuclear contours, and fine chromatin (400x). Immunohistochemically, neoplastic cells were focally positive for CD34 (d, 200x) and CD61 (e, 200x). f) blastic cells with fine chromatin, prominent nucleoli, basophilic cytoplasm, and prominent cytoplasmic blebs seen in the aspiration smear (Giemsa, 1000x)

While waiting for NGS results, the patient received three courses Paclitaxel (200 mg/m2) + Carboplatin (5 mg AUC) + Bevacizumab (15 mg/kg) therapy every three weeks with the presumptive diagnosis of malignant mesenchymal tumor. There was partial response at the end of third chemotherapy course. Since the patient’s splenomegaly and cytopenia did not respond well to the treatment, another bone marrow biopsy was performed. Microscopic examination of this biopsy revealed a hypercellular bone marrow with spindle cell infiltration in a fibrotic background (Fig. 3a-b-c). Immunohistochemically, neoplastic cells were focally positive for CD34 (Fig. 3d) and CD61 (Fig. 3e) and negative for TdT, CD3, CD20, and CD68. In the aspiration smears, more than 20% of the cells were blasts with fine chromatin, multiple prominent nucleoli, basophilic cytoplasm, and cytoplasmic blebs (Fig. 3f). Myeloid precursors showed dysplastic features. The morphology of blasts and the presence of CD61 expression were suggestive of megakaryoblastic differentiation. In the meantime, an RNA sequencing study revealed the presence of *RBM15::MRTFA(MKL1)* fusion.

Based on the evaluation of all these findings together, acute myeloid leukemia with *RBM15::MRTFA(MKL1)* fusion diagnosis was made, and the diagnosis for soft tissue lesion was revised to myeloid sarcoma.

## Discussion

Acute myeloid leukemia with *RBM15::MRTFA(MKL1)* fusion, which is the consequence of t(1;22)(p13;q13), is a molecularly defined distinctive subtype of AML with megakaryoblastic differentiation [2]. It is usually seen in infants without Down syndrome and presents with anemia, thrombocytopenia, and hepatosplenomegaly. Bone marrow biopsy generally shows extensive fibrosis with increased reticulin fibers and interpretation can be hard because of the crush artifact, but typical megakaryoblasts might be appreciated in the bone marrow aspiration or peripheral blood smears [1]. Megakaryoblasts show immunopositivity for one or more platelet glycoproteins like CD41, CD61, and CD42b [2]. Detection of *RBM15::MRTFA(MKL1)* fusion by FISH, RT-PCR, etc. is essential for a definite diagnosis. Extramedullary involvement known as myeloid sarcoma is reported in the liver, spleen, lymph nodes, lung, bone, and pancreas in this patient group [1, 8, 10–12]. Samples from extramedullary sites can mimic non-hematopoietic malignancies like neuroblastoma [8, 9], hepatoblastoma [10], or small round blue cell tumors [11] due to extensive fibrosis, the cohesiveness of epithelioid/spindle-looking neoplastic cells, the presence of vascular/sinusoidal invasion or shared immunophenotypic features. Therefore, atypical extramedullary tumors in the pediatric age group should be evaluated along with bone marrow biopsy. Utilization of advanced molecular techniques would also be helpful in difficult cases.

In our case, the interpretation of the first bone marrow biopsy was suboptimal to reach a clear diagnosis due to a prominent crush artifact, even though a few blastic cells suggestive of megakaryoblastic differentiation were observed in the aspiration smears. Excisional biopsy from the right thigh lesion showed cohesive tumor cells with epithelioid to spindled morphology in a fibrotic background, vascular involvement, and immunopositivity for vascular markers (CD31, CD34, ERG, and factor VIII). These findings led to the misdiagnosis of malignant vascular neoplasm. However, the second bone marrow biopsy that consisted of more than 20% megakaryoblasts and detection of *RBM15::MRTFA(MKL1)* fusion by RNA sequencing in the soft tissue lesion has prompted a diagnosis of AML with *RBM15::MRTFA(MKL1)* fusion. As a consequence, the diagnosis of the soft tissue lesion was amended to myeloid sarcoma. *RBM15::MRTFA* is among the recurring translocations defining acute myeloid leukemia. In the presence of this recurrent genetic abnormality, more than or equal to 10% blast count is required for AML diagnosis in the ICC 2022 classification [13]. However, a diagnosis of acute myeloid leukemia can be rendered regardless of blast count in the WHO 2022 classification if *RBM15::MRTFA* can be demonstrated with genetic studies. Therefore, genetic studies are especially valuable in cases, such as ours, where significant bone marrow fibrosis is present. Significant bone marrow fibrosis, which usually accompanies AMKL can prevent obtaining sufficient bone marrow aspirate to enumerate blast counts reliably, highlighting the importance of clinicopathological suspicion and molecular tests for the diagnosis.

Myeloid sarcoma is described as a tumor mass composed of myeloid blasts involving any anatomical sites other than bone marrow. Skin and soft tissue are reported as more frequently involved sites in children [6, 7]. It has been known that myeloid sarcoma shares similar molecular and cytogenetic alterations with bone marrow AML [7]. To the best of our knowledge, in the literature, there are only a few cases of AMKL with *RBM15::MRTFA(MKL1)* fusion that presented with myeloid sarcoma [8–11]. In our patient, biopsy-proven soft tissue involvement and radiologically detected skin, liver, spleen, lymph node, and lung involvements were considered as evidence of extramedullary disease. Samples from extramedullary sites, without proper bone marrow sampling, can lead to misdiagnosis of non-hematopoietic neoplasm as in our case. Therefore, in children with atypical soft tissue lesions, the possibility of myeloid sarcoma should be considered in the differential diagnosis, especially if there is evidence of bone marrow involvement.

The process leading to a misdiagnosis of malignant vascular tumor, particularly composite hemangioendothelioma, a vascular tumor composed of a complex admixture of histologically benign and malignant vascular components, is ameliorated by the complex morphology and immunohistochemical expressions of CD31, CD34, ERG, and Factor VIII, largely known as vascular/endothelial markers [14]. Morphologically, the tumor was composed of monotonous histiocytoid spindle cells resembling an intermediate sarcoma and nested or papillary-like involvement of vascular spaces, reminiscent of papillary intralymphatic angioendothelioma. However, the clinical setting especially extensive involvement of bone marrow and hepatosplenomegaly is not compatible with the diagnosis of composite hemangioendothelioma. Among the vascular markers, CD34 and ERG are the least specific for endothelial cells; the former is expressed in many mesenchymal tumors and the latter is also found in epithelioid sarcomas and prostatic adenocarcinomas. Although CD31 and Factor VIII are known to be more specific to endothelial cells, CD31 expression in macrophages, megakaryocytes, and platelets [15, 16] and Factor VIII expression in platelets [17] constitute the major pitfall misleading to an erroneous preliminary diagnosis of vascular tumor in this case. Given the discordance of clinical and pathological findings, utilization of a non-targeted molecular test, e.g. RNA sequencing, was essential to reach the correct diagnosis.

The prognosis of AML with *RBM15::MRTFA(MKL1)* fusion is not fully clear. Some studies suggest a better prognosis [12, 18] while others suggest a worse prognosis [19, 20] compared with other AMKL subtypes. Analysis of the prognosis of the reported cases with extramedullary involvement reveals that two of the patients died before the correct diagnosis was made [9, 10], one died within 11 months due to disease progression [11], and one was in complete remission in the second year of diagnosis [8]. We started the BFM AML 2019 protocol, and he achieved clinical and radiological remission after one cycle of induction. A clinical decision will be made whether the patient will undergo allogeneic stem cell transplantation or not, depending on the response to subsequent treatment.

In conclusion, AML with *RBM15::MRTFA(MKL1)* fusion can initially present as a soft tissue lesion in children and may lead to misdiagnosis of the malignant mesenchymal tumor. It would be wise to remember myeloid sarcoma in atypical extramedullary lesions in pediatric patients.

## Data Availability

Data sharing not applicable to this article as no datasets were generated or analysed during the current study.

## Declarations

This is a case report with depersonalized data of the patient and did not require informed consent. Verbal and written consent has also been obtained.
